# Prevalence of Type 2 Diabetes Mellitus and Its Microvascular Complications in Sri Lanka: A Systematic Review and Meta-Analysis

**DOI:** 10.7759/cureus.95630

**Published:** 2025-10-28

**Authors:** Harahana Munasinghe, Sithara Wijekoon, Pansujee Dissanayaka, DDM Jayasundara, Mapa Prabhath Piyasena, Sobha Sivaprasad, M D Nugawela

**Affiliations:** 1 Department of Statistics and Computer Science, University of Kelaniya, Gampaha, LKA; 2 Department of Statistics, Queenland University of Technology, Brisbane, AUS; 3 Vision and Eye Research Institute, School of Medicine, Anglia Ruskin University, Cambridge, GBR; 4 Department of Ophthalmology, Moorfields Eye Hospital, London, GBR; 5 Institute of Ophthalmology, University College London, London, GBR

**Keywords:** complications of diabetes, diabetes prevalence, meta-analysis, sri lanka, systematic review

## Abstract

The burden of type II diabetes and microvascular complications is increasing around the globe, especially in low- and middle-income countries. We conducted a systematic review and a meta-analysis to estimate the prevalence of type II diabetes and its microvascular complications to determine the nature of diabetes and its complications in Sri Lanka. A systematic review and meta-analysis were conducted by searching PubMed, EMBASE, and CINAHL for studies published between 2014 and 2024 on the prevalence of type II diabetes mellitus and its microvascular complications in Sri Lanka. Titles, abstracts, and full texts were screened against predefined inclusion/exclusion criteria, and data were extracted from eligible studies. Pooled prevalence estimates were calculated using a random effects model, with 95% confidence intervals, and results were presented using forest plots. Analyses were performed using Stata version 18 (StataCorp LLC, College Station, Texas, USA). A total of 1400 articles underwent title, abstract or full text screening and only 11 (0.8%) articles were left for the analysis, which satisfied the inclusion/exclusion criteria; 15839 people with type II diabetes mellitus (mean sample size n=2263 (SD 2374), with a mean age of 48.6 years (SD 4.94)), were included. The estimated overall prevalence of type II diabetes, diabetic retinopathy and neuropathy was 17% (95% CI 17% to 18%), 13% (95% CI 13% to 14%) and 19% (95% CI 16% to 21%), respectively. The forest plots illustrated variation across individual studies but also showed a consistent upward trend in the prevalence of diabetes over time, with more recent studies reporting higher estimates. Further research into cost-effective methods for early identification and treatment of people with diabetes is needed to reduce the associated morbidity and mortality. Screening for complications at the time of diagnosis should become a routine practice to provide rapid intervention.

## Introduction and background

Diabetes mellitus is a chronic metabolic disease that occurs when the pancreas does not produce sufficient insulin or when the body cannot effectively utilise the insulin it produces [1]. According to the International Diabetes Federation (IDF) Diabetes Atlas 2025, approximately 629 million people worldwide are living with diabetes, making it a major cause of morbidity and mortality. This number is projected to rise to 783 million by 2045, with the greatest burden falling on low- and middle-income countries (LMICs) [2].

Diabetes can be classified into type I diabetes, type II diabetes, and gestational diabetes [2]. Among these, type II diabetes mellitus (T2DM) accounts for the majority of the cases globally, which is the focus of this study. Over 90% of people with diabetes have T2DM, driven by socio-economic, demographic, environmental, and genetic factors [2].

T2DM leads to a range of complications, broadly classified into microvascular and macrovascular conditions. The main microvascular complications are diabetic retinopathy, nephropathy, and neuropathy (including diabetic foot disease), while macrovascular complications include cardiovascular and cerebrovascular disease [3,4]. Diabetic retinopathy is the most common microvascular complication, and sight-threatening diabetic retinopathy has become a leading cause of visual impairment worldwide, particularly among working-age adults [2,5]. These complications contribute substantially to diabetes-related morbidity and mortality. While both microvascular and macrovascular complications impose a significant health burden, the present review focused exclusively on microvascular complications.

Globally, several systematic reviews and meta-analyses have examined the prevalence of diabetes and its complications [6 -10]. In 2021, global diabetes prevalence among people aged 20 to 79 was estimated as 10.5% [6]. The global prevalence of diabetic retinopathy over the last decade (2015-2025) was around 27%, but it's higher in Africa, where it can reach up to 35%, and in Southeast Asia, where it can go as high as 40% [7]. A meta-analysis conducted in 2020 estimated that the prevalence of neuropathy among people with diabetes was 30%, with a 95% confidence interval ranging from 25% to 34% [8]. A review conducted in 2023 using 20 studies found that the overall rate of diabetic nephropathy among people with diabetes was 27% [9].

When considering the global prevalence of diabetes, the Southeast Asia region has become one of the fastest-growing areas in the world for diabetes cases. Countries like India, Bangladesh, and Sri Lanka are playing a big role in this trend, mainly because of urbanisation, population ageing, and changes routines of their daily lives [2].

In Sri Lanka, the burden of diabetes has grown significantly. According to the Department of Census and Statistics (DCS) of Sri Lanka, in 2016, approximately 8.4% of adults aged 15 years and above had self-reported that they had been diagnosed with diabetes by a clinician [11]. In 2019, the crude prevalence of diabetes was estimated as 23% [12]. According to IDF projections, about 1.2 million adults were estimated to have diabetes in Sri Lanka in 2025, a number expected to rise to 1.7 million by 2045 [2]. The prevalence is higher among urban residents, women, more affluent groups, and Muslim adults [12].

However, most epidemiological studies have been conducted in high-income countries, limiting their relevance to LMICs [13]. Despite this increasing burden, there is a lack of recent systematic reviews focused specifically on T2DM and its microvascular complications in Sri Lanka. Previous reviews have been limited: a 2012 regional review of South Asia included only two Sri Lankan studies [13]; a 2015 review identified urban residency and family history as major risk factors [14]; and a 2023 review reported a prevalence of 12.07% [15], but its pooling of studies spanning two decades may have underestimated the rising trend over time.

Given these gaps, a comprehensive review of T2DM and its microvascular complications in Sri Lanka is warranted. Such evidence will provide an updated understanding of the national burden and support the development of context-specific strategies for prevention, early detection, and management.

Accordingly, our study aimed to systematically review the prevalence of T2DM and its microvascular complications among the adult population in Sri Lanka.

## Review

Materials and methods

This study protocol (PROSPERO CRD42021278060) and the methods of reporting results adhered to the principles of Meta-Analysis of the Preferred Reporting Items for Systematic Reviews and Meta-Analyses (PRISMA) and the Cochrane guidelines. Funnel plots were used to estimate the publication bias of the meta-analysis. Two authors (HM and PD) independently searched all the published manuscripts, including the titles, abstracts, and full articles for potentially eligible studies. 

Study Design

The CoCoPop framework of meta-analysis of observational studies was used as the study design. Sri Lankans aged above 18 years suffering from T2DM were included. Observational studies reported in English that were published during the last 10 years, specifically for the period 2014 - 2024, were only included. A detailed description of the study protocol is shown in Table 1.

**Table 1:** Study protocol of the systematic review

CoCoPop	Inclusion Criteria	Exclusion Criteria
Condition	Type II diabetes mellitus and microvascular complications, namely, nephropathy, neuropathy, retinopathy and diabetic foot.	Persons with complications due to type I diabetes and self-reported diabetes
Context	Sri Lankans who are diagnosed with type II diabetes	Countries other than Sri Lanka
Population	Sri Lankans aged > 18 years diagnosed with type II diabetes mellitus with or without microvascular complications.	Persons suffering from type 1 diabetes and gestational diabetes. Children aged ≤ 18 years suffering from diabetes and microvascular complications
Outcomes	Prevalence of type II diabetes and prevalence of microvascular complications in Sri Lanka.	Investigate the management of the diabetes patients. Investigate the infrastructure of the hospitals and the human resources.
Study Design	Observational Studies	Interventional Studies

Electronic Database Search

The search was done on PubMed, EMBASE, and CINAHL from January 2014 to December 31st, 2024. The keywords that were used to search were: (((diabetes) OR (diabetic)) OR Neuropathy OR Nerve Damage OR Eye Problems OR retinopathy OR nephropathy OR Kidney problems) AND (Sri Lanka). Abstract screening and full-text screening were conducted by two authors (HM and PD) independently. Discrepancies during the screening process were resolved through consultation with a third reviewer (MD). The reference lists of retrieved studies were also reviewed for other relevant studies. 

Definitions of Diabetes Mellitus and Complications

The definitions used to identify people with diabetes in the reviewed studies are listed in Table 2.

**Table 2:** Definition of the complications of the diabetes ADA: American Diabetes Association

Complication	Definition
Retinopathy	Diabetic retinopathy was defined as signs of retinal ischemia and/or increased retinal vascular permeability, with loss of vision due to neo-vascularization, hemorrhage, retinal detachment, macular edema and/or retinal capillary non-perfusion [16].
Neuropathy	According to the ADA, neuropathy is characterized as nerve damage that occurs throughout the body [16].
Nephropathy	According to the Medscape definition, nephropathy is defined as persistent albuminuria (>300 mg/d or >200 μg/min) that is confirmed on at least two occasions 3-6 months apart [16].

Type II Diabetes

In this review, T2DM was defined in accordance with the 2024 American Diabetes Association (ADA). Fasting blood glucose (FBG ≥7.0 mmol/L), two-hour plasma glucose (2h-OGTT ≥11.1mmol/L), glycated hemoglobin (≥48mmol/mol (6.5%)) and a random plasma glucose (≥ 11.1 mmol/L) for the patients with classic symptoms of hyperglycemia or hyperglycemic crisis were the reported diagnostic parameters [17].

Data Extraction

Two independent reviewers (HM and SW) extracted data from the included articles. The extracted information includes: (1) basic information such as title, first author, publication year, country/region, study period, and study design; (2) baseline characteristics: sample size, gender, average age, exposures, and diagnosis; (3) prevalence results: prevalence and 95% CIs of microvascular complications of T2DM.

Quality Assessment

The risk of bias in the included articles was simultaneously evaluated, and any disagreements were resolved through discussion or were determined by a senior author, if necessary (MN). Joanna Briggs Institute's (JBI) critical appraisal checklist was used to assess the methodological quality of the included cross-sectional studies [18,19]. Each paper was assessed using the eight questions designed to check the quality of the studies, in which each question could be answered with yes, no, unclear, or not applicable.

Statistical Analysis

Prevalence measures for T2DM, retinopathy and neuropathy were presented using forest plots. Clinic-based studies and population-based studies published during the period 2014-2024 were considered as two subgroups to identify the trend over time in the prevalence of T2DM and microvascular complications. Pooled prevalence of subgroups and the overall prevalence were presented in the forest plots. A random effects meta-analysis model was used to estimate the pooled prevalence in subgroups and in overall prevalence. The risk of publication bias was assessed qualitatively and quantitatively using a funnel plot. Statistical software Stata version 18 (StataCorp LLC, College Station, Texas, USA) was used during the statistical analysis.

Results

Study Selection

A total of 1400 studies published up to December 31st, 2024, were extracted using the keyword search. Of these studies, after title, abstract, and full text screening, only 11 articles (Table 3) were left for the analysis, which satisfied the inclusion/exclusion criteria [5,12,20-28].

**Table 3:** Included articles in the review

	Author & Year	Study Design	Number of Participants	Gender Distribution		Mean Age (with SD)	Prevalence of Diabetes Mellitus (DM) DR & Neuropathy (%)
				Male	Female		
1	Rannan-Eliya et al. 2023 [12]	Cross-sectional study	6661	3265	3396	50.1 (17.2)	DM=23.0
2	Katulanda et al. 2015 [20]	Cross-sectional study	4482	1770	2712	37.1(5.9)	DM=11.9 DR= 27.5
3	Somasundaram et al. 2019 [21]	Cross-sectional study	463	139	324	58.3 (10.3)	DM=27.6
4	De Silva et al. 2018 [22]	Cross-sectional study	1234	628	606		DM=16.3
5	Gamlath et al. 2017 [23]	Cross-sectional study	1011	408	603		DM=19.0
6	Herath et al. 2017 [24]	Cross-sectional study	1470	756	714	50.5 (17.6)	DM=16.3
7	Amarasinghe et al. 2015 [25]	Cross-sectional study	518	224	294		DM=16.4
8	Katulanda et al. 2014 [5]	Cross-sectional study	684	246	438	37.1(5.9)	DR = 18.1
9	Kaluarachchi et al. 2020 [26]	Cross-sectional study	334	89	245	58.2 (10.6)	DR= 33.2 Neuropathy = 34.1
10	Liyanage et al. 2018 [27]	Cross-sectional study	482	122	360	60.7 (10.5)	DR=21.4 Neuropathy =13.1
11	Sujanitha et al. 2015 [28]	Cross-sectional study	8401	4293	4108	60.9 (11.3)	DR= 12.0

**Figure 1:**
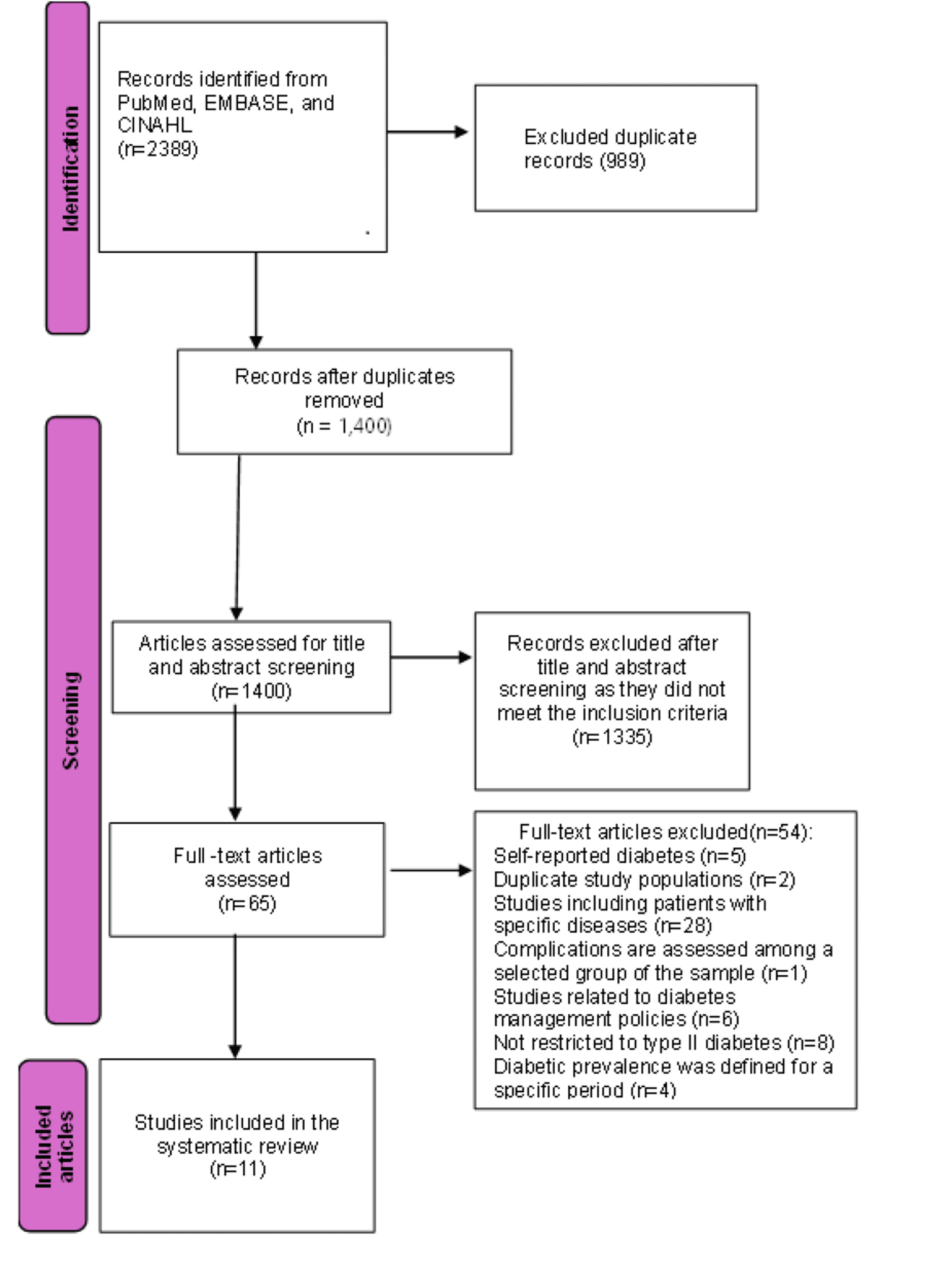
PRISMA flow chart PRISMA: Preferred Reporting Items for Systematic Reviews and Meta-Analyses

The screening process is shown using the PRISMA flow diagram (Figure 1). As we limited our study to the period 2014-2024, our study results consist of studies from 2014 to 2023. Studies were excluded if they relied on self-reported diabetes status, included patients with specific comorbidities, did not represent the target population, examined other conditions, or were conducted outside the defined study period. Out of the 11 articles included in the analysis, seven studies reported the prevalence of T2DM [12,20-25], five studies on diabetic retinopathy [5,20,26-28], and two on diabetes-related neuropathy [26,27], with some overlap between the studies. Nine studies were clinic-based cross-sectional studies, whereas only two were population-based. Due to the heterogeneity among the studies, subgroup meta-analysis was conducted on the prevalence of T2DM, diabetic retinopathy and neuropathy. We could not find any study on diabetic nephropathy that was conducted during the period 2014-2024 due to a lack of publications on nephropathy.

Quality Assessment of the Studies

The quality of the enrolled studies was assessed with the JBI critical appraisal checklist (Table 4). The study subjects and setting were described in detail in all 11 studies; criteria for inclusion in the sample, objective, and criteria used to measure the exposure (diabetes status) were mentioned in detail in most of them. However, some of them did not use appropriate statistical methods (for example method of sampling) for analysis, and most of them did not use appropriate strategies to identify and adjust for confounding factors.

**Table 4:** Joanna Briggs Institute's (JBI) Checklist for quality assessment Q1 = Were the criteria for inclusion in the sample clearly defined?, Q2 = Were the study subjects and the setting described in detail?, Q3 = Was the exposure measured in a valid and reliable way?, Q4 = Were objective, standard criteria used for measurement of the condition?, Q5 = Were confounding factors identified?, Q6 = Were strategies to deal with confounding factors stated?, Q7 = Were the outcomes measured in a valid and reliable way?, Q8.= Was appropriate statistical analysis used?

Study ID	Author & Year	JBI Checklist Questions								Exclude/Include
		Q1	Q2	Q3	Q4	Q5	Q6	Q7	Q8	
1	Katulanda et al. 2014 [5]	✓	✓	✓	✓	X	X	✓	✓	Include
2	Katulanda et al. 2015 [20]	✓	✓	✓	✓	X	X	✓	✓	Include
3	Liyanage et al. 2018 [27]	X	✓	✓	✓	X	X	✓	✓	Include
4	Somasundaram et al. 2019 [21]	✓	✓	✓	✓	X	X	✓	✓	Include
5	Sujanitha et al. 2015 [28]	✓	✓	✓	✓	X	X	✓	✓	Include
6	Gamlath et al. 2017 [23]	✓	✓	✓	✓	X	X	✓	✓	Include
7	Amarasinghe et al. 2015 [25]	✓	✓	✓	✓	X	X	✓	X	Include
8	De Silva et al. 2018 [22]	✓	✓	✓	✓	X	X	✓	X	Include
9	Herath et al. 2017 [24]	✓	✓	X	X	X	X	✓	✓	Include
10	Kaluarachchci et al. 2020 [26]	✓	✓	X	X	X	X	✓	✓	Include
11	Ranan-Eliya et al. 2023 [12]	✓	✓	✓	✓	X	X	✓	✓	Include

Meta-Analysis Results

**Figure 2:**
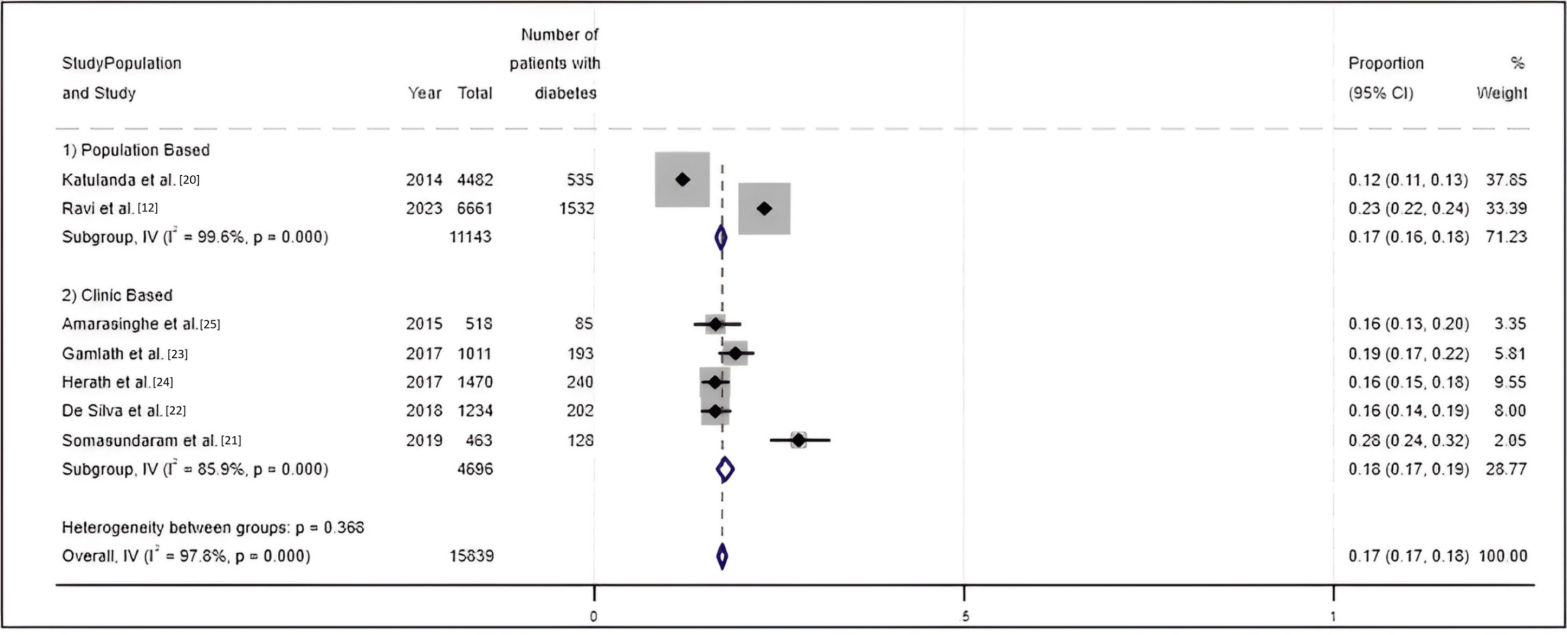
Forest plot for prevalence of diabetes

Meta-analysis results for the diabetes prevalence: Seven studies reported results for the prevalence of T2DM among Sri Lankans. These studies used random samples from cross-sectional studies. The forest plot (Figure 2) highlights that the prevalence of type II diabetes mellitus ranged from 12% to 28% during the last 10 years (2014-2024) in Sri Lanka [12,20-25].

There were two population-based studies [12,20]. Among them, one was conducted by Katulanda et al. in 2015, using a sample of 4482 individuals aged 20 and above from seven provinces of Sri Lanka [20]. In this study prevalence of T2DM was estimated as 12%. In the most recent study conducted by Rannan-Eliya et al. in 2019, which included 6661 participants who were above 45 years of age, the crude prevalence of diabetes was reported as 23.0% (95% CI 21.2% to 24.7%) [12].

In contrast, most of the clinic-based cross-sectional studies have estimated the prevalence of T2DM using sample sizes varying from 450 to 1500 participants with a relatively high prevalence of T2DM (28%) [21] due to purposive sampling.

The forest plot has estimated the overall prevalence of T2DM as 17% (95% CI 17% to 18%) with a high level of heterogeneity (I^2^ = 97.8). The results suggest a possible increasing trend in diabetes prevalence over time in both clinic-based and population-based studies; however, this interpretation should be made with caution, given the high heterogeneity among included studies, which may reflect differences in study design, population characteristics, and diagnostic criteria. When only clinic-based studies were considered, the pooled prevalence increased to 18% (95% CI 17% to 19%), with reduced, but still high, heterogeneity (I^2^ = 85.9) (Figure 2).

**Figure 3:**
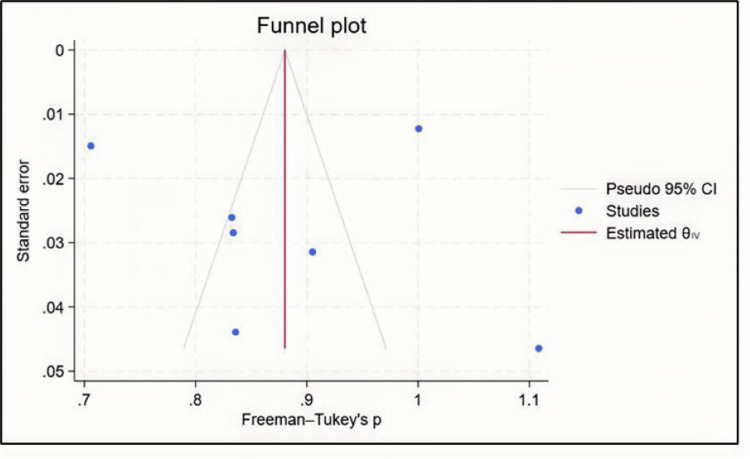
Funnel plot for prevalence of diabetes [12,20-25]

According to Figure 3, the funnel plot is clearly asymmetric, indicating publication bias.

**Figure 4:**
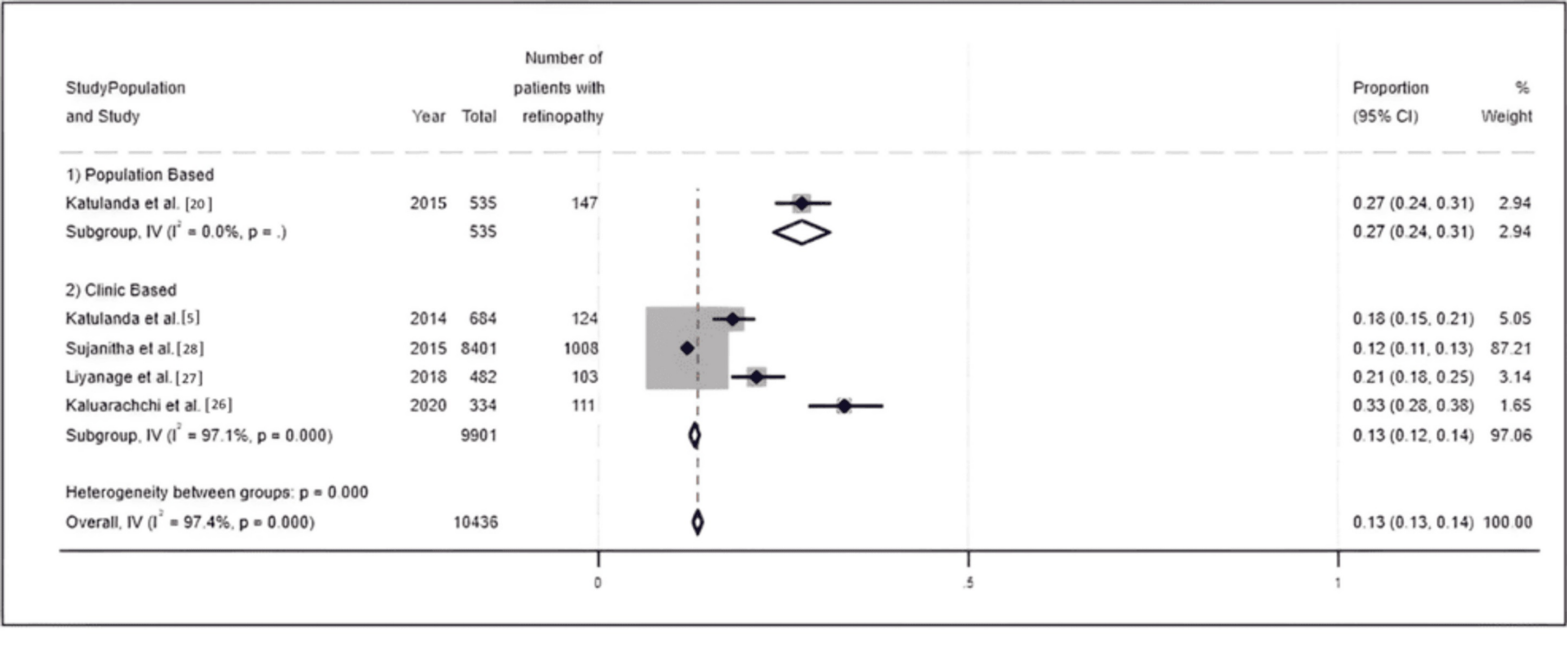
Forest plot for the prevalence of diabetic retinopathy

Meta-analysis results for the prevalence of diabetic retinopathy: For diabetic retinopathy, data were extracted from five studies conducted between 2014 and 2024 [5,20,26-28]. During this period, the prevalence of diabetic retinopathy varied from 12% to 33%. However, the highest prevalence of diabetic retinopathy reported within the time frame was around 30% with Kaluarachchi et al. reporting 33% in 2020 [26] and Katulanda et al. reporting 27% in 2015 [5]. Overall prevalence of diabetic retinopathy was estimated as 13% (95% CI 13% to 14%) with a high level of heterogeneity (I^2^ = 97.4) (Figure 4).

**Figure 5:**
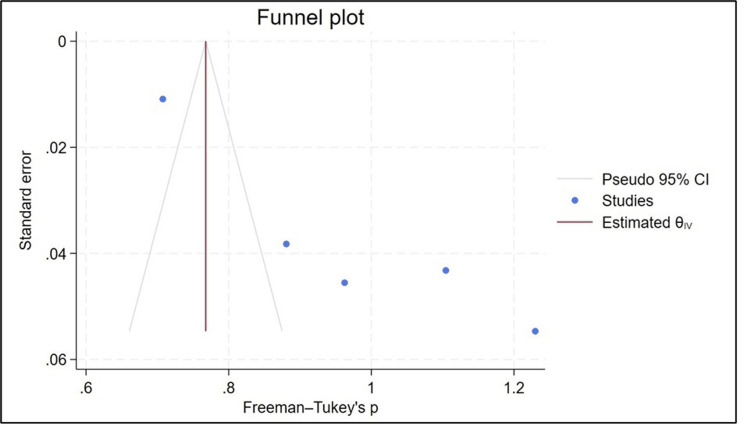
Funnel plot for the prevalence of retinopathy [5,20,26-28]

The funnel plot of the studies was produced. According to Figure 5, the funnel plot is asymmetric, highlighting the presence of publication bias.

**Figure 6:**
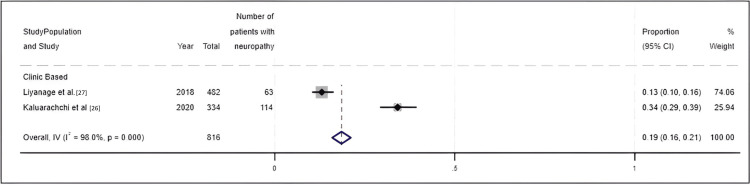
Forest plot for the prevalence of neuropathy

Meta-analysis results for the prevalence of neuropathy: In relation to diabetic neuropathy, two articles presented data on prevalence that ranged from 13% to 34%. According to the meta-analysis, the prevalence of neuropathy has increased over the years, with an overall prevalence of 19% (95% CI 16% to 21%) (Figure 6).

**Figure 7:**
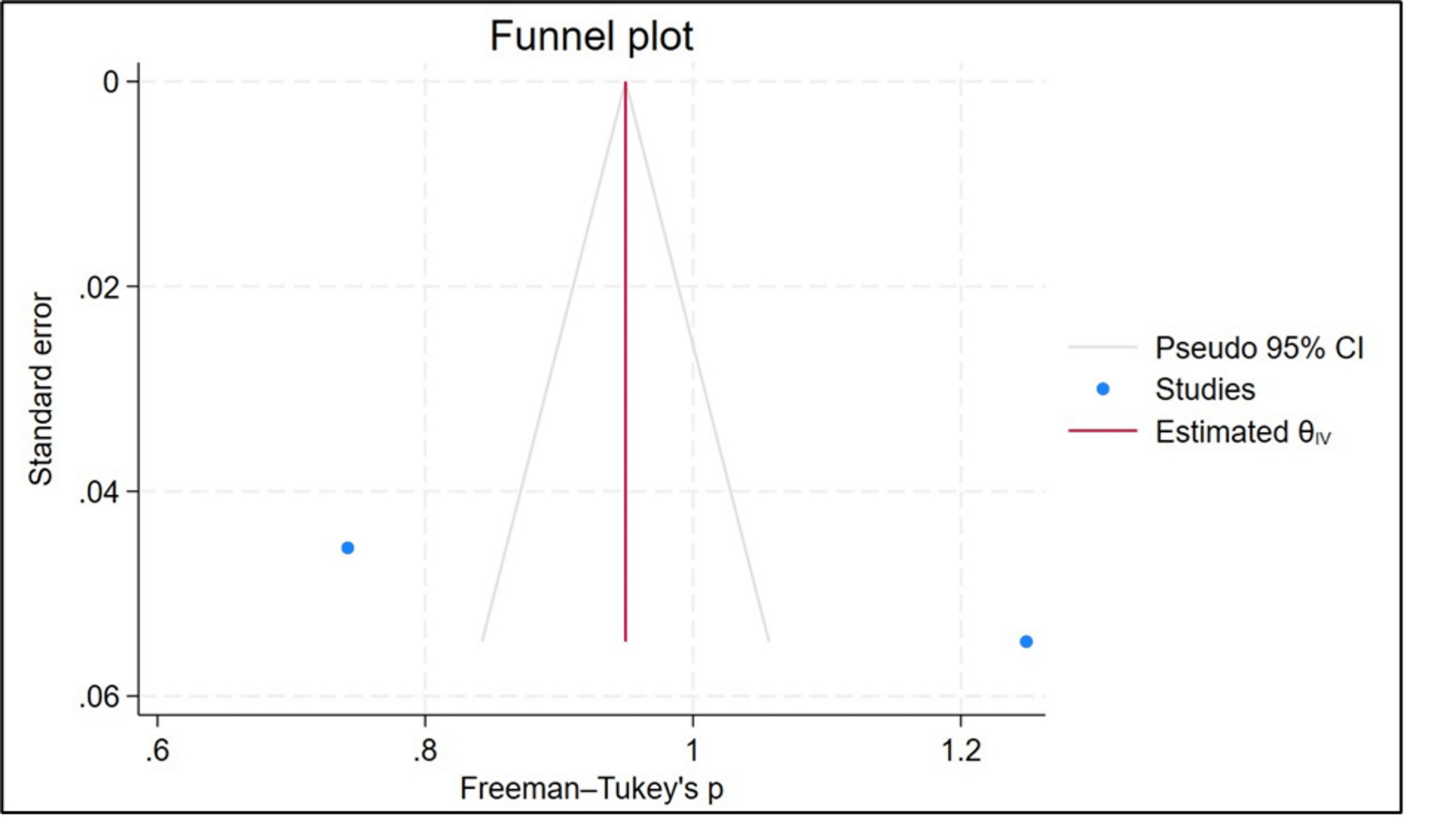
Funnel plot for the prevalence of neuropathy [26,27]

The funnel plot of the studies was produced. According to Figure 7, the funnel plot is asymmetric, highlighting the presence of publication bias.

Discussion

The purpose of this comprehensive review was to gather available data during the last 10 years (2014-2024) on the prevalence of T2DM and microvascular complications among the adult population of Sri Lanka. Hence, the information provided in this review may contribute to important implications for healthcare policy and practice, highlighting the need to develop targeted strategies to reduce the public health burden due to T2DM and microvascular complications in the country.

We identified two studies conducted using nationally representative population-level data on diabetes prevalence during the last 10 years (2014-2024) [12,20]. Most of the remaining studies were clinic-based; however, the similarity in prevalence estimates between clinic- and population-based studies suggests that these findings may still provide a reasonable reflection of the broader population, though caution is warranted when generalizing. In this context, our review underscores the need for more population-based studies to systematically evaluate the prevalence and microvascular complications of diabetes in Sri Lanka. This systematic review and meta-analysis also demonstrated an increasing trend in the prevalence of T2DM. According to Rannan-Eliya et al., the crude prevalence of all types of diabetes was estimated at 23%, while the age-standardized prevalence (using FPG only) was 17% [12], which aligns closely with our overall prevalence estimate of the meta-analysis.

In relation to diabetic retinopathy, we noted an increasing trend in diabetic retinopathy in Sri Lanka. According to Katulanda et al. in 2015, the prevalence of any degree of diabetic retinopathy was 27.4% while a study conducted by Kaluarachchi et al. reported the prevalence of diabetic retinopathy as 33% in 2020 [20,26]. This emphasizes that the prevalence of diabetic retinopathy is increasing over time in Sri Lanka. In relation to diabetic neuropathy, there were only two clinic-based studies. The overall prevalence showed similar results to a study conducted by Katulanda et al. using a national-level dataset in 2012, in which the prevalence of diabetic neuropathy was reported as 24% [29]. The pooled prevalence of diabetic retinopathy in our study was higher than that in studies of countries with high incomes, such as the Netherlands (0.7%) [30], South Korea (2.8%) [31], Denmark (6.8%) [32], and in the UK, which is less than 18% [33,34]. It is even higher than the prevalence in low- and middle-income countries [35]. These variations may reflect the differences in screening and diagnostic methods in each health system and variations in individual factors. Similarly, the estimated pooled prevalence of neuropathy was higher than the prevalence of high-income countries and it is similar to the prevalence in low- and middle-income countries [36].

We were unable to conduct a meta-analysis on diabetic nephropathy due to the limited number of eligible publications meeting the inclusion criteria. Only a small number of studies reported nephropathy-specific prevalence data, and those that did varied considerably in diagnostic definitions, assessment methods, and study populations. This lack of consistent, high-quality data prevented robust pooling of estimates and limited our ability to draw reliable conclusions about the national burden of diabetic nephropathy [36].

There is a recent systematic review that has looked at the current evidence available for diabetes prevalence. However, they have only focused on diabetes prevalence but not on complications. Another limitation of this recent review is that they have presented a combined measure for the diabetes prevalence in Sri Lanka for the period between 1990 and 2022 [15]. However, diabetes prevalence is increasing over time globally as well as in Sri Lanka [2,12]. Given the difficulty of pooling prevalence estimates across long time spans, this study focused on the most recent decade (2014-2024) to ensure greater consistency and relevance of the findings.

Our study has many strengths. When compared with the recently published review on diabetes prevalence in Sri Lanka, our study considered not only T2DM prevalence, but also microvascular complications of diabetes. In addition to that it includes the most recent and the largest national level study which was conducted by Rannan-Eliya et al. in 2019 [12]. Hence our review provides more up-to-date estimates on the prevalence of T2DM in Sri Lanka. The rigorous search strategy we used to explore the eligible studies has increased the quality of included studies. The methodological quality of all included studies was assessed using an appropriate quality appraisal criterion.

Despite these strengths, this study has several limitations. We were unable to assess the influence of individual risk factors such as ethnicity, geographic location, and income level on T2DM and its microvascular complications, as most included studies did not report these variables. Consequently, we could not perform subgroup analyses based on these risk factors. In addition, there was a limited number of population-based studies, while several were clinic-based with relatively small sample sizes and unclear contextual information. Furthermore, all included studies were cross-sectional, which limits the ability to infer temporal or causal relationships.

Furthermore, this meta-analysis of T2DM is based on a limited number of articles which represent national-level data. Many studies were conducted in hospital clinics. Only two studies in this review have provided national-level diabetes prevalence in Sri Lanka [12,20]. Therefore, more national-level data are required to make suitable strategies to diagnose individuals living with diabetes and microvascular complications.

## Conclusions

This review presents important information about the prevalence of T2DM and microvascular complications in Sri Lanka. The findings highlight the increasing trend of diabetes and microvascular complications and, therefore, highlight the need for preventive strategies, screening programmes and treatment pathways for diabetes and microvascular complications in the country.
